# Relatedness Need Satisfaction and the Dark Triad: The Role of Depression and Prevention Focus

**DOI:** 10.3389/fpsyg.2021.677906

**Published:** 2021-07-13

**Authors:** Mengqi Xiao, Zhuofan Wang, Xiaoshan Kong, Xiya Ao, Jia Song, Peng Zhang

**Affiliations:** ^1^School of Educational Science and Technology, Guangdong Polytechnic Normal University, Guangzhou, China; ^2^Department of Psychology, School of Teacher Education, Leshan Normal University, Leshan, China; ^3^Department of Psychology, School of Philosophy, Wuhan University, Wuhan, China; ^4^School of Psychological and Cognitive Sciences, Peking University, Beijing, China; ^5^Department of Psychology, Tsinghua University, Beijing, China

**Keywords:** dark triad, relatedness need satisfaction, depression, prevention focus, conditional process model

## Abstract

Dark triad traits are often associated with maladaptive social and interpersonal interactions, such as dishonesty and self-centeredness; thus, it is important to explore predictors of the dark triad in order to better facilitate the reduction of such behaviors. The present study adopted a self-report approach with a total of 5,207 Chinese undergraduate students participated in the study. We found that relatedness need dissatisfaction significantly predicted the presence of dark personalities, which was mediated by prevention focus. Conditional process model analysis found that this mediating effect was stronger when depression levels were lower. Final study results contributed to further understanding predictors of the dark triad. Study limitations and future research directions were also examined.

## Introduction

Given the continued development of personality psychology, as well as a desire to reflect a more comprehensive view of human personalities and relationships among various personality traits, researchers have shifted from analyzing singular personality traits to the studying personality clusters. Along this vein, a new group of antisocial personality traits, the dark triad (DT), has attracted much attention. Within this trait group, there are three subdimensions: Machiavellianism, narcissism, and psychopathy. Machiavellianism emphasizes self-interest and personal interest maintenance, primarily through deception; narcissism reflects self-centeredness, vanity, and self-righteousness; and psychopathy is generally characterized by impulsive behaviors and a lack of empathy and responsibility (Paulhus, 2014; Muris et al., 2017). All three traits lead to a speedy and exploitative lifestyle (Furnham et al., 2013), making this one of the most undesirable personality groups (Snyder et al., 2019).

Numerous studies have shown that the DT is usually linked to maladaptive social and interpersonal interactions, such as dishonesty and self-centeredness (Paulhus, 2014; Muris et al., 2017). Individuals with high levels of the DT will be more selfish and unjust in both economic and social dilemmas (Terri et al., 2015). The results of meta-analysis showed that Machiavellianism and psychopathy in the workplace were negatively correlated with job performance; the DT is more effective than the Big Five in predicting counterproductive work behaviors (Judge et al., 2006; Jane and Lebreton, 2011). In addition, the DT is positively correlated with antisocial behaviors, such as aggression and bullying (Blais et al., 2014); Machiavellians are more likely to engage in immoral behaviors for profit (Kish-Gephart et al., 2010); narcissists prone to cheat to show superiority and rarely feel guilty about it (Brunell et al., 2011); and psychopaths are highly associated with bullying, opposition to authority, substance abuse, and even criminal behaviors (Williams and Paulhus, 2004).

### Relatedness Need Satisfaction and the Dark Triad

From an evolutionary perspective, researchers have associated dark triad (DT) development to the fast life history strategy (LHS) (Csathó and Birkás, 2018). The DT holds adaptive value for individuals under poor living conditions where competition is fierce. In an effort to gain an advantage in the race for survival, certain individuals have developed a fast LHS, which is characterized by risk-taking, a lack of foresight, the need for timely reward and gratification, and the propensity to support exploitation for direct reproductive-related benefits (Gladden et al., 2009; Jonason et al., 2012a; Mcdonald et al., 2012; Holtzman and Donnellan, 2015), which are nearly identical to the key manifestations of the DT. A large number of studies have also found connections between the DT and fast LHS indicators, such as exhibiting more impulsivity, short-term mating tendencies, and lower empathy (Book et al., 2015; Heym et al., 2019).

However, given tremendous social economic progress and development, individual challenges associated with the DT routinely emerge due to its destructive effect on production and interpersonal relations. Therefore, exploring factors that influence the DT may help provide opportunities for effective interventions. Numerous studies have found that humans, as herd animals, have experiences and psychological feelings related to interpersonal relationships that are often important predictors of personality development and motivational strategies (Gross and John, 2003; Events, 2004; Quan et al., 2021), such as relatedness need satisfaction. Studies have shown that relatedness need satisfaction is closely related to the Big Five personality (Church et al., 2012; Chen et al., 2019; Deventer et al., 2019); however, we do not yet know the relationship between relatedness need and the DT. As a group of antisocial personality characteristics, the DT is mainly characterized by maladaptive social and interpersonal interactions, which may undermine interpersonal relationships.

Self-determination theory (SDT), proposed by Deci and Ryan (2000), suggests that relatedness need is a fundamental human psychological need and is also the universal driver by which people seek and maintain enduring, positive, and meaningful social interactions (Baumeister and Leary, 1995). Relatedness need dissatisfaction leads to insecurity, perceived threat, and a greater sense of competition [for a review, see Ryan and Deci (2017)]. This implies that unsatisfied relatedness need has the potential to predict the DT by activating fast-life strategies. When relatedness need is unsatisfied, individuals are often rejected or receive no positive feedback. They may be inclined to take risks in order to obtain immediate benefits and are more likely to ignore or disobey social norms, resulting in speculation and aggression (Guan and Zhou, 2016). In contrast, when relatedness need is satisfied, individuals with more emotional warmth are less likely to have dark personality traits. Hence, we concluded that unsatisfied relatedness need may be associated with the DT, and proposed Hypothesis 1: Unsatisfied relatedness need is a major predicting factor of the DT.

### The Mediating Role of Regulatory Focus Theory

In addition, studies have linked relatedness need satisfaction and regulatory focus through the need-support model. In 1997, Higgins put forward the regulatory focus theory, which includes prevention and promotion focus (Higgins, 1997). In prevention focus, individuals concentrate on safety need when striving to obtain their goals. They are more sensitive to information related to loss, worry about negative outcomes of a situation, and adopt vigilance strategies to avoid loss (Freitas et al., 2005). Promotion focus, on the other hand, is geared more toward the need for growth and positive outcomes when pursuing goals [see, e.g., Higgins and Spiegel (2004) for a review].

Relatedness need satisfaction can also affect the subjective experiences of regulatory focus. To be more specific, the experience of satisfaction of relatedness need makes people subjectively feel both more opportunities for growth and more possibilities for good things to happen, so they tend to pursue relationship growth, ultimately resulting in a promoting focus. On the contrary, individuals who experience unsatisfied relatedness need are more likely to hold a prevention focus because they sense less opportunity for growth and pay more attention to preventing bad things from happening, that is to say, they are driven by loss avoidance. Therefore, relatedness need satisfaction is negatively correlated with prevention focus and positively correlated with promotion focus (Vaughn, 2017).

Further studies found that prevention focus, as an internal motivation of an individual, affects social interactions. Research has suggested that individuals with a high level of prevention focus show more negative behaviors and emotions toward outgroups, as compared with those holding a low level of prevention focus (Shah et al., 2004). This may be because they perceive out-groups as a threat to their own interests. With this in mind, in order to protect their own interests from being violated, these individuals may put others on the opposite side of interests and activate a self-interest schema, thus potentially leading to fraud, control, and manipulation of others in social interactions, which are typical manifestations of the DT (Jonason et al., 2012b). Accordingly, failing to meet relatedness need may predict higher levels of the DT through prevention focus. Conversely, those utilizing a promotion focus pay more attention to positive outcomes, thus are associated with lower levels of the DT. Therefore, we propose Hypothesis 2: Regulatory focus mediates the relationship between unsatisfied relatedness need and the DT in which prevention focus predicts a higher level of the DT, while promotion focus predicts a lower level of the DT.

### Moderating Role of Depression

The effect of depression must also be considered when discussing the mediating role of regulatory focus on the relationship between relatedness need and the DT. Individuals with high levels of chronic depression were found to have lower levels of empathy. These factors make people less able to effectively communicate with others and feel less interpersonal support and warmth, which predicted higher levels of the DT (Carver and Harmon-Jones, 2009; Park et al., 2013; Hao et al., 2017). Most prior studies on the prediction of dark triad traits suggested that emotions play a negligible role; however, since depression is an important predictor of low-motivation levels (Carver and Harmon-Jones, 2009; Pegg and Kujawa, 2020), high levels of depression may lead to more avoidant and less competitive behaviors (Carver and Harmon-Jones, 2009; Park et al., 2013; Hao et al., 2017), thus weakening the predictive effect of prevention focus on the DT. Accordingly, we propose Hypothesis 3: The mediating effect of regulatory focus on the relationship between relatedness need satisfaction, and the DT is moderated by depression, where the mediating effect is weaker, the higher depression levels are.

**Figure 1:**
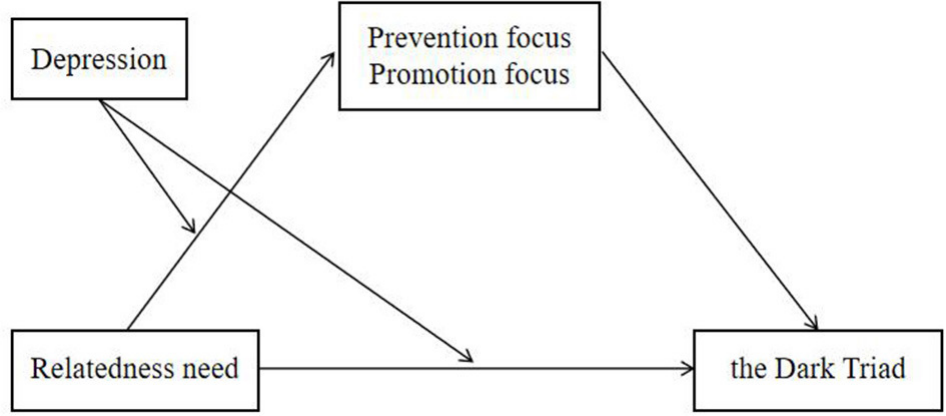
The proposed moderated mediation model.

The data in the current study are part of a large sample of college students. We used the dirty dozen (DD), the relatedness subscale of the basic need satisfaction in general scale (BNSG-S), the Chinese version of the CES-D, and the regulatory focus questionnaire, as well as established a conditional process model to test our hypotheses (Figure 1).

## Method

### Participants

The current study issued questionnaires *via* the Credamo online survey platform by randomly inviting 17- to 22-year-old college students to participate in this study. A total of 5,586 Chinese undergraduate students from three universities in Lanzhou and Gansu provinces were successfully recruited online. The final sample comprised 5,207 students (1,328 females and 3,879 males; *Mage* = 18.95 years, *SD* = 1.00 years; age range: 17–22 years), as 379 students were excluded; 90 participants did not meet the age criteria, 241 completed the survey in less than 300 s, and 48 did not meet the definition of the variables after filling in the missing values, using regression methods. The effective response rate was 93.2%.

### Measures

#### Relatedness Need Satisfaction

We used the relatedness subscale of the basic needs satisfaction in general scale (BNSG-S), which was revised by Xie et al. (2012), to measure relatedness need satisfaction. This scale is an eight-item measure that includes relatedness need satisfaction (five items, e.g., “I feel the person I care about also cares about me”) and relatedness need satisfaction disrupted (three items, e.g., “I felt excluded from the group I wanted to fit in”). The participants were instructed to indicate how true they felt each statement was of their life and respond, using a scale from 1 (Not at all true) to 5 (Very true). Higher scores indicate a higher level of relatedness need satisfaction. In this study, Cronbach's alpha was 0.767.

#### Depression

A short version of Center for Epidemiologic Studies Depression Scale (CES-D-13) (Zhang and Li, 2011) is comprised of 13 questions, including three dimensions: physical symptoms (five items, e.g., “I find it hard to do everything”), depressed mood (five items, e.g., “I feel lonely”), and positive emotions (three items, e.g., “I feel hopeful about the future”). This measure was scored on a four-point scale (1= “none,” 2= “1 to 2 days,” 3 = “3 to 4 days,” and 4 = “more than 5 days”), where the total depression score was calculated through the addition of physical symptoms and depressed mood scores with the reverse score of a positive mood. Higher depression scores on the CES-D-13 indicate more depressive symptoms. Cronbach's alpha coefficient was 0.905.

#### Regulatory Focus

Regulatory focus questionnaire (RFQ) (Higgins et al., 2001), revised by Yao et al. (2008), is a 10-item questionnaire, containing two dimensions: prevention focus (four items, e.g., “While growing up, I always did things my parents could not stand”) and promotion focus (six items, e.g., “I did a good job of what I wanted to do”). The questionnaire was scored on a five-point Likert scale (1 = “never,” 5 = “always”) and contained four reverse-scoring questions. Higher scores indicate a higher level of either prevention or promotion focus. Cronbach's alpha coefficient for prevention focus was 0.711.

#### The Dark Triad

Dirty Dozen (DD), developed by Jonason et al. (2010) and revised by Geng et al. (2015), is a 12-item scale, comprising three dimensions: Machiavellianism (four items, e.g., “I tend to control people to get what I want”), psychopathy (four items, e.g., “I lack a heart of regret”), and narcissism (four items, e.g., “I wish to be praised”). We instructed the participants to contemplate how true they felt each statement was of their life and respond on a scale from 1 (“Does not suit me very well”) to 7 (“Suits me very well”). Higher scores indicate a higher level of the DT. Cronbach's alpha on the total scale was 0.838. Cronbach's alpha of Machiavellianism was 0.900. Cronbach's alpha of psychopathy was 0.544. Cronbach's alpha of narcissism was 0.851.

#### Control Variables

According to descriptive analysis, correlations show that gender, age, and family socioeconomic status (SES) were associated with the DT. Therefore, we included these variables as covariates in all analyses. We created a composite index of SES by averaging standardized values of occupation income and education levels of their parents (Xu et al., 2009; Yuan et al., 2009; OECD, 2012).

### Procedure

We administered this study, using an online questionnaire in which we first presented an informed consent form to the participate before initiating the formal questionnaire portion. After indicating consent, the participants were then required to provide demographic information, including gender, age, and education level. Next, they completed a series of self-report questionnaires, including Dirty Dozen, the regulatory focus questionnaire, the relatedness subscale of the basic needs satisfaction in general scale, and the CES-D-13. Upon completion, the participants were thanked and received 20 RMB (~$3USD) in compensation. All the procedures were approved by the Ethics Committee of Psychological Research, Guangdong Polytechnic Normal University.

### Statistical Analyses

The study used SPSS 25.0 to process and analyze data. First, descriptive statistics and correlation analyses were conducted on study variables. Then, to explore how relatedness need satisfaction affects the development of DT personalities, we conducted a conditional process model test, using the PROCESS macro program in SPSS developed by Hayes (2012).

Initially, we handled the missing data with regression, and then we computed the SES score. In order to control the common method variance (CMV), we used the single method-factor approach recommended by Xiong et al. (2012) to further test the common method variance. Second, we calculated descriptive statistics for both variables of interest and control variables, followed by bivariate associations among these variables. Third, we further examined whether depression moderated the mediation process. The analysis of the moderated mediation model was performed by using the PROCESS macro of model 8 of Hayes (2013). Gender, age, and SES were used as covariates.

## Result

### Common Method Variance

In this study, we conducted strict control procedures in order to facilitate the self-reporting mechanism we used to collect data. For example, we uniformly adopted any anonymous surveys, and all scales held high reliability and validity. There were also several questions which used reverse scoring. After finishing data collection, we used the single method-factor approach recommended by Xiong et al. (2012) in order to control common method variance (CMV) and further test common method deviation. To analyze factor structure, we constructed a confirmatory factor analysis as Model 1, and then constructed Model 2, which contained common method factors. We compared the fitting indexes of Model 1 and 2. Changes in these fit indexes indicate that the model was significantly improved by adding the common method factor (Δχ^2^/df = 27.43, ΔCFI = 0.047, ΔTFI = 0.066, ΔSRMR = 0.032, ΔRMSEA = 0.042). As a result, there is no obvious common method variance in the measurement.

### Descriptive Analyses

Table 1 presents the means, standard deviations, and correlations between variables. All variables were significantly correlated in the expected direction. Relatedness need satisfaction was negatively correlated with the DT, depression, and prevention focus but was positively correlated with promotion focus. Furthermore, depression, prevention focus, and the DT were positively correlated with one another, while promotion focus was positively correlated with the DT.

**Table 1:** Summary of means, standard deviations, and correlations of variables of interest.

	***M***	***SD***	**1**	**2**	**3**	**4**	**5**	**6**	**7**
1 Gender	1.26	0.44							
2 Age	18.95	1.00	−0.053^**^						
3 SES	−0.01	0.97	−0.042^**^	−0.119^**^					
4 R-need satisfaction	29.84	4.93	−0.044^**^	0.023	0.013				
5 Depression	23.19	7.87	0.099^**^	0.001	0.059^**^	−0.592^**^			
6 Prevention focus	9.52	2.66	−0.082^**^	−0.052^**^	0.108^**^	−0.422^**^	0.447^**^		
7 Promotion focus	18.46	2.03	−0.077^**^	0.009	−0.006	0.360^**^	−0.387^**^	−0.259^**^	
8 The Dark Triad	38.38	11.04	−0.119^**^	−0.058^**^	0.130^**^	−0.415^**^	0.416^**^	0.413^**^	−0.179^**^

*R-need satisfaction, relatedness need satisfaction.*

*N = 5,207, gender was dummy coded such that 1 = male and 2 = female.*

*M, mean; SD, standard deviation.*

** *p < 0.01*.

### Testing for Moderated Mediation

The results of correlation analysis indicate that the model can be tested for both mediating and moderating effects. Furthermore, the correlation analysis indicated that the two subdimensions of regulatory focus did not predict DT personality in the same direction, and that the two variables were verified separately for whether they played a mediating role in the prediction of relatedness need satisfaction for the DT. Mediation analyses revealed that the promotion focus mediated the association between relatedness need satisfaction and the DT; however, the mediating effect was very weak [3%, β = −0.0124, *SE* =0.0053, CL = (−0.0228, −0.0020)], so we no longer included this promotion focus in our analysis. We used Model 59 of the PROCESS macro (Hayes, 2013) to analyze the moderating effect, but our results suggest that depression only moderated the direct effect and the first half of the mediation model. Next, Model 8 was used to analyze the moderating effect of depression and the mediating effect of prevention focus, while gender, age, and SES were used as covariates.

The results illustrated that relatedness need satisfaction significantly negatively predicted prevention focus (β = −0.245, *p* < 0.001), and that prevention focus significantly positively predicted the DT (β = 0.207, *p* < 0.001). Additionally, the mediation effect of prevention focus is significant. The interaction of relatedness need satisfaction and depression had a significant predictive effect on the DT and prevention focus (the dark triad: β = 0.042, *t* = 4.075, *p* < 0.001; prevention focus: β = 0.042, *t* = 4.223, *p* < 0.001); that is to say, depression can moderate the predictive effect of relatedness need on dark personality and prevention focus (Table 2).

**Table 2:** Regression testing of the moderated mediation model.

**Predictors**	**Model 1 (Prevention focus)**			**Model 2 (The Dark Triad)**		
	**β**	***SE***	***t***	**β**	***SE***	***t***
Gender	−0.281	0.028	−10.228^***^	−0.300	0.027	−10.980^***^
Age	−0.044	0.012	−3.688^***^	−0.040	0.012	−3.387^***^
SES	0.083	0.012	6.860^***^	0.088	0.012	7.412^***^
R-need satisfaction	−0.245	0.015	−16.581^***^	−0.213	0.015	−14.273^***^
Depression	0.328	0.016	21.066^***^	0.226	0.016	14.166^***^
R-need satisfaction × Depression	0.042	0.010	4.075^***^	0.042	0.010	4.223^***^
Prevention focus				0.207	0.014	15.178^***^
*R^2^*	0.240			0.540		
*F*	311.789^***^			305.041^***^		

*R-need satisfaction, relatedness need satisfaction.*

*All variables in the model were standardized before the regression.*

*SE, standard error; 95% CI, confidence interval with lower and upper limits.*

*** *p < 0.001*.

**Figure 2:**
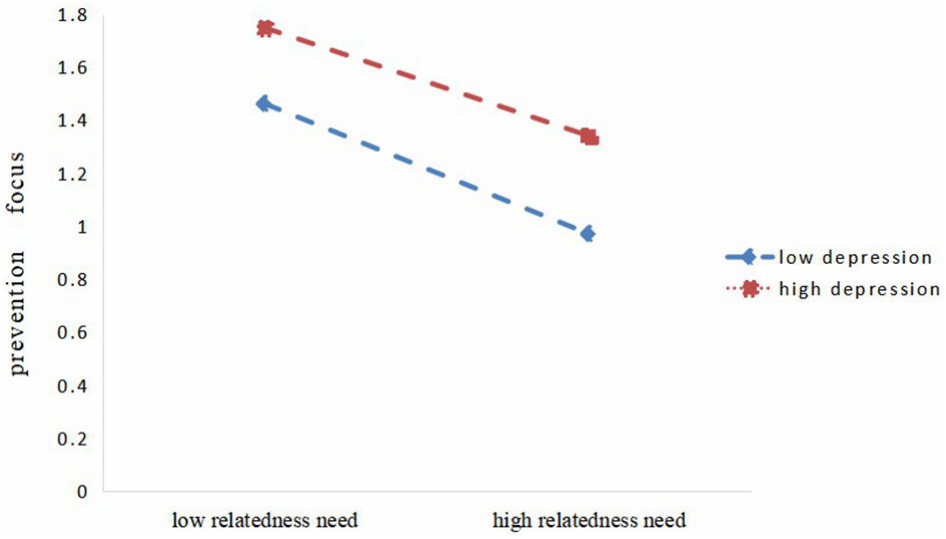
Depression moderates relatedness need satisfaction and prevention focus.

Furthermore, simple slope analysis showed that the relatedness need was a stronger predictor of prevention focus in subjects with low levels of depression (*M*-1*SD*), in comparison to participates with high levels of depression (*M*+1*SD, simple slope* = −0.213, *t* = −11.92, *p* < 0.001) (Figure 2). Mediating effect analysis showed that the mediating effect of prevention focus diminished with increasing levels of depression (see Table 3).

**Table 3:** Mediating effects and direct effects on different levels of depression.

	**Depression**	**β**	**Boot*SE***	**BootLLCI**	**BootULCI**
Prevention focus	*M*-1*SD*	−0.287	0.018	−0.323	−0.251
	*M*	−0.245	0.015	−0.274	−0.216
	*M*+1*SD*	−0.204	0.018	−0.239	−0.169
The Dark Triad	*M*-1*SD*	−0.255	0.018	−0.291	−0.219
	M	−0.213	0.015	−0.242	−0.184
	*M*+1*SD*	−0.171	0.018	−0.205	−0.136

*CI, confidence interval with lower and upper limits*.

**Figure 3:**
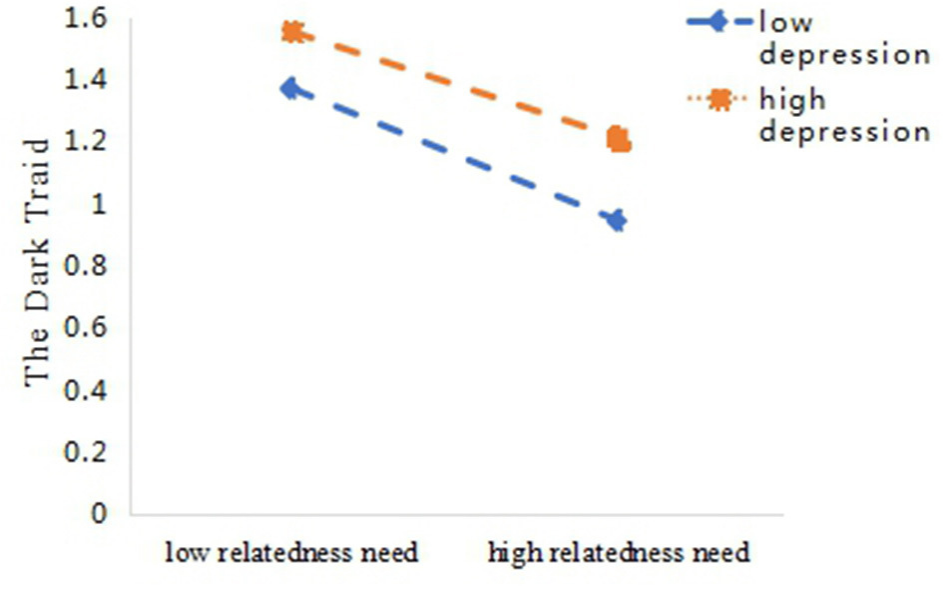
Depression moderates relatedness need satisfaction and the dark triad.

Additionally, compared with high levels of depression (*M*+1*SD, simple slope* = −0.213, *t* = −11.92, *p* < 0.001), relatedness need satisfaction in participants with low levels of depression (*M*-1*SD, simple slope* = −0.314, *t* = −17.26, *p* < 0.001) had a stronger predictive effect on the DT (Figure 3). Data analysis revealed that the direct effect component of the mediating effect of relatedness need to meet predicted DT personality diminished with increasing levels of depression (Table 3).

## Discussion

Our study investigated the relationship between relatedness need satisfaction, regulatory focus, and the DT by constructing a conditional process model, taking into consideration the effects of SES and depression.

Our results show that relatedness need satisfaction can negatively predict the DT, and that this relationship can be mediated by prevention focus. At the same time, the mediating effect was dampened by higher levels of depression. To our knowledge, this is the first study to examine the relationship between these variables. Our study found that relatedness need satisfaction was negatively correlated with three subdimensions of the DT: narcissism, psychopathy, and Machiavellianism, verifying Hypothesis 1. Negative consequences of relatedness need dissatisfaction, such as insecurity and social threats, are all able to activate the fast LHS (Belsky et al., 1991; Brumbach et al., 2009). Under the circumstances, individuals show characteristics of a lack of foresight, need for timely interests, a tendency toward exploitation, and lower levels of empathy, which are all important characteristics of the DT.

Further data analysis found that prevention focus played a partial mediating role between relatedness need satisfaction and the DT, while the mediating effect of promotion focus was very weak, partially verifying Hypothesis 2. Previous studies found that relatedness need satisfaction can positively predict promotion focus and negatively predict prevention focus (Vaughn, 2017). This may be due to a perception that out-groups pose a threat to interests of an individual, which is thought to be an important factor in activating the fast LHS. Based on this, individuals may cheat, control, and manipulate others during social interactions in order to protect their own interests or compete for resources, which are typical manifestations of the DT (Jonason et al., 2012b). Thus, relatedness need dissatisfaction may predict higher levels of the DT through prevention focus, which is consistent with Hypothesis 2. Meanwhile, relatedness need satisfaction is correlated with lower levels of the DT, because it leads to promotion focus, which makes individuals more focused on positive outcomes. Senses brought by relatedness need satisfaction, such as security, interpersonal trust, and cooperative tendency, can directly lead to adaptive social interactions and are thus less affected by the behavioral motivation system. Therefore, we failed to find a mediating effect of promotion focus on relatedness need to predict dark personality.

Considering the significant effect of emotion on the motivation system, we investigated the moderating role of depression in the above-mediating relationship. Results showed that the mediating effect was significantly weaker at higher levels of depression, which verified Hypothesis 3. We thought this may be due to low-motivation levels correlated with depression. The present study found that relatedness need dissatisfaction and the DT were significantly positively correlated with depression, which was consistent with prior research (e.g., Ibarra-Rovillard and Kuiper, 2011; Shih et al., 2019). Depression may have a competitive effect when prevention focus, functioning as a mediating variable, serves as a motivation system and may activate the fast LHS, given that depression serves as an important predictor of low motivation levels (Carver and Harmon-Jones, 2009; Pegg and Kujawa, 2020). In a state of high depression, the prediction of unsatisfied relatedness need for the DT may be correlated with decreased empathy levels (e.g., Schreiter et al., 2013).

In addition, a major feature of our study is the large sample size. As a negative personality trait, the DT can weaken the influence of representativeness bias and social desirability with a large sample size.

### Implications

In conclusion, our research revealed how relatedness need satisfaction predicts the DT through prevention focus and found that this effect diminished in cases of high levels of depression, which may help us better understand the development mechanisms of the DT. This suggests that the maladaptive social interactions represented by the DT traits are closely related to relatedness need satisfaction of an individual. As social animals, human beings have deep evolutionary roots in social needs (Buss, 1995). If individuals do not feel adequate interpersonal security, they tend to perceive others as a threat and initiate more primitive and competitive fast-life strategies. Our study provides new evidence to the evolutionary roots of the DT.

The results also showed us that timely relatedness need satisfaction is an important protective factor in adaptive personality development, although it may only be effective in people with low levels of depression. Relatedness need satisfaction can reduce prevention focus and thus predict lower levels of the DT, which is achieved through the regulation of the behavioral motivation system. However, when depression levels are high, the role of the motivation system is weakened, and depression may directly predict the DT through emotional-related pathways. This prompted us to consider the influence of variables, such as depression, that may influence the level of motivation in studies involving motivation systems (Roseman, 2013).

### Limitations and Prospects

This study also carries certain limitations. First, although our sample size is large, all the participants were college students, so researchers should be careful with generalizing our research conclusions toward other groups. For example, some studies have shown that age and political experience are related to the DT (Barlett and Barlett, 2015). Thus, it is a worthy topic to explore the influence of different life experiences on the DT personality from a developmental perspective.

Second, some studies have pointed out that the brevity of the dirty dozen psychopathy subscale may have been obtained at the expense of construct validity (Miller et al., 2012). To be specific, from a five-factor model (FFM) perspective, psychopathy comprises a high degree of interpersonal antagonism, insufficient conscientiousness, and a mixture of anxiety and depressions on traits related to neuroticism (e.g., high anger; low anxiety) and extraversion (e.g., high assertiveness; low warmth). However, there is an important variance related to interpersonal antagonism and disinhibition that is not assessed by the DD. Additionally, after analyzing various Machiavellianism measurement scales, researchers have identified four facets, which are manipulation, morality, detachment, and cynicism. However, the DD scale primarily captured manipulativeness (Truhan et al., 2021). Thus, the DD provides an assessment of specific facets of Machiavellianism, vice a comprehensive measurement of all domains. Therefore, the results obtained by using DD in this study may not fully and accurately reflect the nature of the DT.

Third, the present study did not directly measure the level of motivation of the participants, which may weaken the reliability of our conclusions. Since the self-reported regulatory focus is a strategic motivation, vice a motivation level, future research should include investigations of motivation levels and actual behavioral tendencies. This understanding could further clarify the threshold at which the emotion and motivation systems compete against one another, when relatedness need satisfaction predicts the DT.

Last, the current study did not take into account compensation strategies when relatedness need is unsatisfied. Studies have found that when relatedness need is not satisfied, individuals may adopt various differing strategies in order to cope with social rejection. Prevention focus is an example of a negative strategy, while positive strategies also exist, such as the pursuit of meaning in life (Mead et al., 2011). Future studies can also explore the effects of different coping strategies on predictors of relatedness need satisfaction and the DT, which will be helpful in understanding the influence of strategy selections on personality development.

## Conclusions

1. Relatedness need satisfaction has a negative predictive effect on the dark triad;
2. Relatedness need cannot only directly predict the dark triad but also indirectly influence it through prevention focus, which plays a partial mediating role between relatedness need and the dark triad;
3. Depression plays a moderating role in the above-mediation model. When depression levels are high, the mediating effect is weakened.

## Data Availability Statement

The original contributions presented in the study are included in the article/supplementary material, further inquiries can be directed to the corresponding author/s.

## Ethics Statement

The studies involving human participants were reviewed and approved by the Ethics Committee of Psychological Research, Guangdong Polytechnic Normal University. The patients/participants provided their written informed consent to participate in this study.

## Author Contributions

All authors listed have made a substantial, direct and intellectual contribution to the work, and approved it for publication.

## Conflict of Interest

The authors declare that the research was conducted in the absence of any commercial or financial relationships that could be construed as a potential conflict of interest.
